# Rethinking Cognitive Interventions in Bipolar Disorder: Feasibility and First Insights From Metacognitive Group Training (MCT‐Bipolar)

**DOI:** 10.1002/cpp.70294

**Published:** 2026-06-07

**Authors:** Antonie Schoenleber, Esther Quinlivan, Lena Jelinek, Franziska Miegel, Felix Bermpohl, Steffen Moritz, Stefanie Schreiter, Eva Friedel

**Affiliations:** ^1^ Charité – University Medical Center Berlin, Corporate Member of Freie Universität Berlin and Humboldt‐Universität zu Berlin, Department of Psychiatry and Neurosciences, Charité Campus Mitte Berlin Germany; ^2^ Department of Psychotherapy and Psychiatry University Medical Center Hamburg‐Eppendorf Hamburg Germany; ^3^ Charité – University Medical Center Berlin, Corporate Member of Freie Universität Berlin and Humboldt‐Universität zu Berlin, Department of Psychiatry and Psychotherapy, Charité at St. Hedwig Hospital Berlin Germany

**Keywords:** bipolar disorder, dropout, feasibility, linear mixed model, metacognitive training, satisfaction

## Abstract

The feasibility of psychological interventions should be considered when developing and evaluating new therapy methods. Indicators of low feasibility may be a high dropout rate, a low attendance rate and low treatment satisfaction, which threaten the validity of clinical research studies. Given the severity and chronicity of bipolar disorder and its impact on psychosocial functioning, effective treatments are needed to alleviate depressive and manic symptoms. The study evaluated the feasibility and preliminary treatment effects of a novel disorder‐specific metacognitive group training for bipolar disorder (MCT‐Bipolar), which targets cognitive biases specific to depressive and manic episodes while also incorporating mindfulness techniques. Forty‐eight outpatients with bipolar disorder I and II participated in the training conducted by a clinical psychologist for eight consecutive weeks in groups of six to 10 people. Inclusion criteria were euthymia or mild or moderate depressive symptoms. Predictors of dropout and attendance rates were analysed using regression methods, and secondary exploratory analyses examined changes in various efficacy indicators between baseline, 1 week after training and 6 months after training using linear mixed models. In total, 10 participants (20.8%) dropped out, while completers attended an average of six out of eight sessions. Treatment satisfaction of the completers was high. Linear mixed models revealed an increase in mindfulness from baseline to follow‐up. Results on psychosocial functioning showed an increase from baseline to post‐training, though this effect disappeared at the 6‐month follow‐up. Findings support the feasibility of MCT‐Bipolar and suggest potential benefits for mindfulness and short‐term psychosocial functioning. Further research with larger samples and a control group is needed to assess its efficacy.

**Trial Registration:** The evaluation study is registered in the German Registry of Clinical trials (DRKS, registration number DRKS00035704) and received approval from the ethical committee of Charité – University Hospital Berlin (No. EA1/076/22).

## Summary

Metacognitive Training for Bipolar Disorder (MCT‐Bipolar) appears to be a feasible and well‐accepted group intervention, showing high treatment satisfaction and good attendance among participants. It may enhance mindfulness skills and short‐term psychosocial functioning, making it a promising adjunct to standard care. Clinicians may consider MCT‐Bipolar a low‐threshold, group‐based treatment option for individuals with bipolar disorder, although further evidence on clinical efficacy is needed.

## 1 Introduction

Bipolar disorder (BD) can be described as a complex group of often severe and chronic mental disorders characterized by recurring episodes of depression and (hypo)mania shown through extreme fluctuations in a person's mood and energy level (American Psychiatric Association 2017; Berking and Rief 2012). The primary treatment approach for BD consists of pharmacotherapy, complemented by structured psychoeducation, evidence‐based psychological interventions (e.g., cognitive behavioural therapy [CBT] and family‐focused therapy) and adjunctive treatment such as systematic mood monitoring to control symptoms and maintain treatment success (DGBS e.V and DGPPN e.V 2019; Yatham et al. 2018). While individual CBT has proven to be effective in reducing depressive and manic symptoms and supporting patients in preventing relapses and increasing their overall functionality (Chiang et al. 2017), research on group‐based interventions has yielded mixed results. Although studies on extensive group psychoeducation have suggested effectiveness in terms of relapse prevention (Colom, Vieta, Martinez‐Aran, et al. 2003; Colom, Vieta, Reinares, et al. 2003; Castle et al. 2010), two broader meta‐analyses examining psychological interventions, including psychoeducation, CBT, mindfulness and social cognition and interaction training, and dialectical behaviour therapy for adults with BD, rated the overall evidence of psychological group interventions to be of low quality and described the impact on depressive symptoms and hospital admissions as inconclusive (Oud et al. 2016; Tan et al. 2022). Given that the high demand for therapy currently exceeds the capacity of the German healthcare system (BPtK 2023), group therapy may offer a practical alternative by treating multiple patients simultaneously. In this context, Haffner and colleagues were the first to do a pilot study on the effects of a ‘metacognitive training for bipolar disorder’ (MCT‐Bipolar) as a novel disorder‐specific psychological group training that targets patients' metacognitive abilities (Haffner et al. 2018). Originally developed in 2007 by Moritz and Woodward (Moritz and Woodward 2007) for psychosis, MCT aims to raise awareness of maladaptive cognitive biases underlying psychotic symptoms. A recent meta‐review by Meinert et al. demonstrated the effectiveness of MCT in reducing delusion, hallucinations, cognitive biases and negative symptoms, suggesting MCT is an evidence‐based and effective approach in treating psychosis (Meinhart et al. 2025).

While well documented in schizophrenia, maladaptive metacognitive skills may be relevant in the development and persistence of BD (van Camp et al. 2019; Alloy et al. 2009). Based on the Self‐regulatory Executive Function (S‐REF) Model by Wells and Matthews (1996), researches have shown that patients with unipolar depression and BD exhibited significantly more negative beliefs about worry concerning uncontrollability and danger as well as beliefs about the need to control thoughts than healthy control groups (Sarisoy et al. 2014). Additionally, Lahera et al. (2012) found that, even during periods of sustained remission or euthymia, patients with depression exhibited deficits in social cognition, impacting overall psychosocial functioning. To address these problems, Jelinek et al. (2016) developed the Metacognitive Training for Depression (D‐MCT), which targets dysfunctional metacognitive coping strategies (e.g., rumination, thought suppression), thought patterns (e.g., overgeneralization, jumping to conclusions) and cognitive biases that are specific to depression (e.g., black‐and‐white thinking). Because studies on D‐MCT have yielded positive and sustained results regarding depression severity and dysfunctional cognitive and metacognitive beliefs (Jelinek et al. 2016; Jelinek et al. 2019; Hauschildt et al. 2022), Haffner et al. (2018) developed and evaluated MCT‐Bipolar as an adapted version of MCT for psychosis and depression. MCT‐Bipolar integrates elements of psychoeducation, CBT and mindfulness training due to the positive effect of mindfulness‐based cognitive therapy in BD (Deckersbach et al. 2012; Weber et al. 2017).

In a pilot study on MCT‐Bipolar, the 30 participants who met the inclusion criteria (diagnosis of BD, current euthymia and impaired social functioning) took part in a group training that was divided into eight weekly sessions and was conducted by clinical psychologists with expertise in the treatment of BD. In the pre‐post comparison, the results revealed significant large improvements in psychosocial functioning with a large effect size of *r* = 0.52 (*p* = 0.001) but no improvement in depressive and manic symptoms, which was likely due to low baseline levels (Haffner et al. 2018). While the study provides useful preliminary findings, its interpretation should take into account the relatively small sample size (*n* = 30), the absence of a control group, the exclusion of moderately and severely depressed patients, the absence of follow‐up data and potential confounding factors.

Research suggests that patients' characteristics may influence the success of an intervention, though the evidence on MCT remains inconclusive. For example, in a study on people with first‐time psychosis, MCT was shown to be more effective in reducing symptoms and improving cognitive insight for women than for men (Salas‐Sender et al. 2020). Age‐related factors may also impact the likelihood of a person to benefit from MCT, as has been observed in other psychological treatments for depression (Cuijpers et al. 2020). However, two meta‐analysis examining moderators of MCT for psychosis found no differential effects on symptom reduction as a function of age or gender (Penney et al. 2022; Gelner et al. 2025). In addition, participants' expectations about the treatment may influence the treatment's effect on outcomes (Stinson et al. 2018). As a component of the well‐studied placebo response (Huneke et al. 2025; Schedlowski et al. 2015), positive expectations as well as perceived treatment credibility, defined as the patient's perception of how convincing and logical the treatment seems, have been linked to improvement in psychiatric disorders in numerous studies (e.g., (Mooney et al. 2014; Devilly and Borkovec 2000; Cohen et al. 2015; Constantino et al. 2018; Constantino et al. 2019; Price et al. 2008)).

Moreover, if a psychological intervention is not considered adequate by the individuals it is designed to help, they may be more likely to miss therapy sessions, which in turn can diminish the positive effects of the psychological intervention and threaten the validity of clinical trials (Delgadillo et al. 2014; Holdsworth et al. 2014). Treatment satisfaction can be described as the “extent to which treatment gratifies the wants, wishes and desires of the client for services” (Brunero et al. 2009) and results from expectations on the one hand and outcome on the other (Stinson et al. 2018; Attkisson and Zweck 1982; Linder‐Pelz 1982). Consistent with the described theoretical considerations, symptom reduction is associated with satisfaction in various studies on mental health services (Deen et al. 2011; Stabell et al. 2019; Hundt et al. 2013). Overall, the growing number of studies on treatment satisfaction reflects an increasing awareness of the relevance of considering patients' perspectives in clinical trials: Acuña et al. conducted a systematic review on patient satisfaction with MCT for psychosis underlying the importance of studying user satisfaction for the overall success of an intervention (Acuña et al. 2024). Understanding factors like treatment acceptability and attrition can help tailor interventions to patients' needs and improve trial validity. While Haffner et al. (BPtK 2023) provided initial evidence for the efficacy of MCT‐Bipolar, further research is needed with larger, more diverse samples and a person‐centred approach.

This study examines the feasibility and preliminary treatment effects of MCT‐Bipolar in a longitudinal pre‐post design. Feasibility is assessed via dropout rate, attendance and treatment satisfaction. Changes in clinical symptoms, psychosocial functioning, metacognition, mindfulness and related outcomes are analysed using linear mixed models (LMM), including potential predictors such as age, gender, attendance rate, satisfaction, expectancy and credibility of the intervention. The goal of this study is to gain information about MCT‐Bipolar to facilitate its implementation and increase the validity of future studies.

## 2 Method

### 2.1 Sample

The enrolment of participants took place in the outpatient centre of the department of psychiatry and neurosciences at Charité–Universitätsmedizin Berlin and the psychiatric university hospital of Charité at St. Hedwig in central Berlin. The eligible candidates were outpatients who were currently in treatment for bipolar disorder I or II, based on the DSM‐5 (American Psychiatric Association 2017). Further inclusion criteria were current euthymia at inclusion (T0), which was defined by a threshold of < 20 points on the Young Mania Rating Scale (YMRS; Bourin et al. 2014), or the existence of mild or moderate depressive symptoms defined by a score of < 15 on the clinical version of the Questionnaire of Depressive Symptom (QIDS‐C; Rush et al. 2003). Excluded from the study were patients who described acute suicidal thoughts, had a diagnosis of schizoaffective disorder, schizophrenia, substance use disorder, antisocial disorder or an organic disorder, or were under 18 or over 75 years old.

### 2.2 Procedure

In an uncontrolled, longitudinal pre‐post design, MCT‐Bipolar, representing an adapted version of MCT for psychosis and depression (Moritz and Woodward 2007; Jelinek et al. 2016), was applied by a clinical psychologist as a weekly intervention in groups of 10 people for eight consecutive weeks. Data were collected and processed using Redcap, a computer‐based data documenting program (Harris et al. 2019; Harris et al. 2009). Questionnaires were administered at different time points throughout the study: 1 week before the first session (T0), after each session (T0.1–T 0.8), 1 week after the last session (T1) and 6 months after the end of the training (T2). The assessment was conducted by a master's student of psychology and supervised by trained psychiatrists and psychotherapists. After the participants had given informed consent to participate in the study and signed a data privacy statement, they were asked questions about sociodemographics as well as their clinical history, their current treatment and their current affective state. Post‐intervention at T1 and T2, the patients were able to fill out the questionnaires online. The study was approved by the local ethics committee (no. EA1/076/22) and registered in the German study register (DRKS; DRKS00035704).

### 2.3 Metacognitive Training

MCT‐Bipolar consists of eight modules, all focusing on different session‐specific topics about cognitive biases, perfectionism, empathy and self‐esteem (all German slides are available for download at uke.de/bipolar; for more details, see Sondergeld et al. 2025). Each of the eight sessions lasted approximately 60–90 min and started with a 10‐ to 20‐min mindfulness exercise. To encourage interactions and to make the intervention easier to follow, the information was presented in PowerPoint slides, incorporating illustrated examples of daily life situations. The training took place in person at the outpatient centre of the Department of Psychiatry and Neurosciences at Charité.

### 2.4 Measures

Information about age, gender, education level, occupation and income was assessed in a structured interview at T0. The participants who did not fill out the questionnaires after the end of the 8‐week training period (T1) were classified as dropouts. Attendance was measured by the number of sessions attended. Satisfaction was assessed using the German version of the Client Satisfaction Questionnaire (CSQ‐8; German version: ZUF‐8; Larsen et al. 1979; Schmidt and Nübling 2002) adapted to the context. Items are rated on a Likert scale from 1 (*low satisfaction*) to 4 (*high satisfaction*). To evaluate safety, the Inventory for the Assessment of Negative Effects of Psychotherapy (INEP; Ladwig et al. 2014) was administered post‐intervention (T1).

The German translation of the Quick Inventory of Depressive Symptoms Self‐Rating (QIDS‐SR; 48) was administered at all time points. The clinical‐rating version (QIDS‐C; 48) was administered at T0, T1 and T2. Both versions assess depressive symptoms over the preceding 7 days and include items targeting the nine DSM‐IV domains of depression. Manic symptoms were measured at all time points by the German translation of the Altman Self‐Rating Mania Scale (ASMR; Altman et al. 1997), a 5‐item scale to evaluate the severity of manic symptoms. Additionally, the clinical‐rated Young Mania Rating Scale (YMRS‐D) (Mühlbacher et al. 2011; Young et al. 1978) was administered at T0, T1 and T2.

The short version of the International Classification of Functioning, Disability and Health (Mini‐ICF‐APP‐S) was administered to self‐assess capacity limitations related to chronic mental disorders. This self‐rating scale was developed in addition to the observer‐rated MINI‐ICF‐APP, an internationally validated tool (Linden et al. 2022; Linden et al. 2018).

A short version of the Metacognition Questionnaire (MCQ‐30; Wells and Cartwright‐Hatton 2004) was administered to measure changes in metacognitive beliefs following the metacognitive concept of Wells (S‐REF model; Wells and Matthews 1996; Wells 2002). Three of the five subscales were hypothesized to be affected by the training's content: ‘Positive Beliefs about Worry’ (PB), ‘Cognitive Self‐Consciousness’ (CSC) and ‘Negative Beliefs about Uncontrollability and Danger of Worry’ (NEG). A total score was calculated for each subscale as well as for the overall scale. Higher scores indicate higher dysfunctional self‐beliefs associated with psychological disorders (Wells and Cartwright‐Hatton 2004).

Quality of life was measured using the WHOQOL‐BREF (THE WHOQOL GROUP 1998). This instrument includes 25 health‐related items grouped into four domains: Social Relations, Physical Health, Psychological Health and Environment. Domain scores (range: 4–20) are calculated by multiplying the mean score of each domain by four. A total score is obtained by averaging the domain scores.

Negative thinking patterns were measured using the short form of the Dysfunctional Attitude Scale (DAS‐18; Forms A and B; Wells and Cartwright‐Hatton 2004). This scale evaluates therapy outcomes related to cognitive patterns common in depression.

Available health‐related resources and self‐management abilities were assessed using the ‘Fragebogen zur Erfassung von Ressourcen und Selbstmanagement’ (FERUS; (Jack 2007)). This 66‐item measure evaluates motivation for change, self‐observation, coping and self‐efficacy.

Mindfulness represents an important part of metacognitive training. Besides an introduction to mindfulness‐based concepts, every session was initiated by a short mindfulness exercise. To analyse intra‐individual changes in mindfulness before and after the study, the ‘Freiburger Fragebogen zur Achtsamkeit’ (FFA; Buchheld and Walach 2001; Walach et al. 2006) was used.

Treatment expectancy and credibility were assessed after the first session of the training in which the treatment rationale and a preview of further content were presented. For this, the expectancy and credibility subscales of the Credibility/Expectancy Questionnaire (CEQ; Devilly and Borkovec 2000) were used. Each subscale is composed of three items asking the participants to rate on a scale of 1 to 9 or give a percentage number of 0 to 100 on how they think and feel that the training will improve their symptoms. Potential confounding variables were assessed before the intervention started. To assess cognitive functions, the Trail Making Test A and B (TMT A and B; Leiter and Partington 1949), the Number‐Symbol‐Test (‘Zahlen‐Symbol‐Test’; ZST; Aster et al. 2006) and the Vocabulary‐Test (‘Wortschatztest’; WST; Lehrl et al. 1971) were used.

### 2.5 Statistical Analysis

To answer the primary research question, descriptive statistics of attendance, satisfaction, dropout rates, as well as negative effects of the training were determined. Predictors of dropout were assessed using logistic regression. Predictors of attendance were assessed using multiple regression.

Secondly, changes in variables over the course of the study were analysed by multiple LMM. The advantage of LMM over using repeated measured analysis of variance (ANOVA) is that it can handle missing observations without deleting the entire case (Brown 2021). Further, between‐ and within‐participants variability can be accounted for by allowing for random effects. In comparison with fixed effects, which represent the average intercept and slope over the course of the study, random effects measure the variability between participants. Thirdly, important covariates may be incorporated into the model to provide a more comprehensive understanding of how the training may influence the outcomes. Drawing from the existing literature and logical considerations, the following time‐invariant covariates were hypothesized to affect the effectiveness of the training and thus added to the model: gender, age, satisfaction, credibility, expectancy, concurrent psychotherapy and number of attended sessions. Assumptions of LMM were checked by visual inspection using graphical tools following the recommendations of Singer et al. (2017). Specifically, residual plots were examined to assess the linearity and homoscedasticity of fixed effects by plotting standardized marginal residuals against both the values of each explanatory variable and the fitted values. In addition, random effects were checked using QQ plots and unit index plots to detect potential outliers. In cases where the residuals deviated from the assumed pattern, the affected response variables were log‐transformed to improve model fit. All data analyses were performed using the statistical software R (R Core Team 2021), with the LMM executed through the ‘lme4’ package (Bates et al. 2015). An alpha level of 0.05 was set for all statistical tests.

## 3 Results

### 3.1 Sample

A total of 48 patients were enrolled and screened at baseline. Six consecutive training groups were realized with 6–10 participants in each group. Demographic and clinical characteristics of the sample are shown in Table 1. Participants were on average 41.2 years old (Mdn = 39.5, SD = 11.92, Range = 22–67) and had experienced onset of bipolar symptoms at the average age of 21.8 years (Mdn = 19.0, SD = 8.84). Number of prior depressive, manic and hypomanic episodes differed widely between the participants, with an average number of depressive episodes of 18.1 (Mdn = 8.5, SD = 23.03, Range = 1–120), average number of manic episodes of 2.54 (Mdn = 1.5, SD = 4.22, Range = 0–20) and average number of hypomanic episodes of 10.3 (Mdn = 4.5, SD = 13.42, Range = 0–60). About 89.6% of the participants reported prior hospital admissions due to symptoms, with an average of 3.4 admissions (Mdn = 2.0, SD = 4.71, Range = 1–30).

**TABLE 1:** Sociodemographic and clinical characteristics at baseline.

Baseline characteristics	*N = 48*	%
Gender		
Female	27	56.2
Male	21	43.8
Relationship status		
Single	20	41.7
Partnered	28	58,3
Highest educational level		
Middle school (Mittelschulabschluss)	1	2.1
High school (Mittlere Reife)	6	12.5
Higher education entrance qualification (Hochschulreife)	41	85.4
Employment status		
Unemployed (incl. Student and retirement)	17	35.4
Employed full time (> 35 h)	17	35.4
Employed part‐time (< 35 h)	14	29.2
Income (Euros/Month)		
< 1.000	10	20.8
1000–2000	11	22.9
2000–3000	14	29.2
> 3000	13	27.1
Diagnosis^a^		
Bipolar Type I	26	54.2
Bipolar Type II	22	45.8
Severity of depression^b^		
None (< 6)	26	54.2
Mild (< 11)	11	22.9
Moderate (< 16)	11	22.9
Current treatment		
Psychotherapy	21	43.8
Psychopharmacological treatment	43	83.3

*Note:* Demographic data were self‐reported and assessed by a structured interview.

^a^ Self‐report.

^b^ Measured by the Quick Inventory of Depressive Symptomatology Clinical Version. Cutoffs derived from Rush et al., 2003.

### 3.2 Feasibility

In total, *n* = 38 participants completed the study, which resulted in a dropout rate of 20.83%. At the 6‐month follow‐up, *n* = 25 (52.08%) participants filled out the questionnaires. Clinical ratings of depressive and manic symptoms were only possible for five participants at T2. Reasons for dropping out during the study were reported by eight participants and included not perceiving any benefit from the training (*n* = 5), time overlaps with other appointments (*n* = 1), depressive episode (*n* = 1) and chronic physical illness (*n* = 1). Logistic regression was used to determine whether dropping out was related to any of the baseline measures. Of all the variables, only low MCQ sum scores (metacognitive beliefs) significantly predicted dropout (*B* = −0.34, *p* = 0.025). Further analysis showed that higher MCQ scores on the subscale ‘Negative Beliefs about Uncontrollability and Danger of Worry’ were a significant predictor for completing the training (*B* = −0.19, *p* = 0.025).

On average, the sample had an attendance rate of 66.4% (including dropouts). One participant dropped out after the first session, and the dropouts completed on average 2.2 sessions (SD = 0.78). The completers attended at least four out of the eight sessions of the training with an average attendance of *M* = 6.11 (SD = 1.30) sessions, which corresponds to an attendance rate of 76.4%. Only six participants attended all eight sessions. The most common reasons stated by the participants for not attending were sickness, vacation or work duties. The attendance rate did not differ significantly between training groups (*F* = 1.64, *p* = 0.17), and no baseline measure was found to predict attendance rate.

**FIGURE 1:**
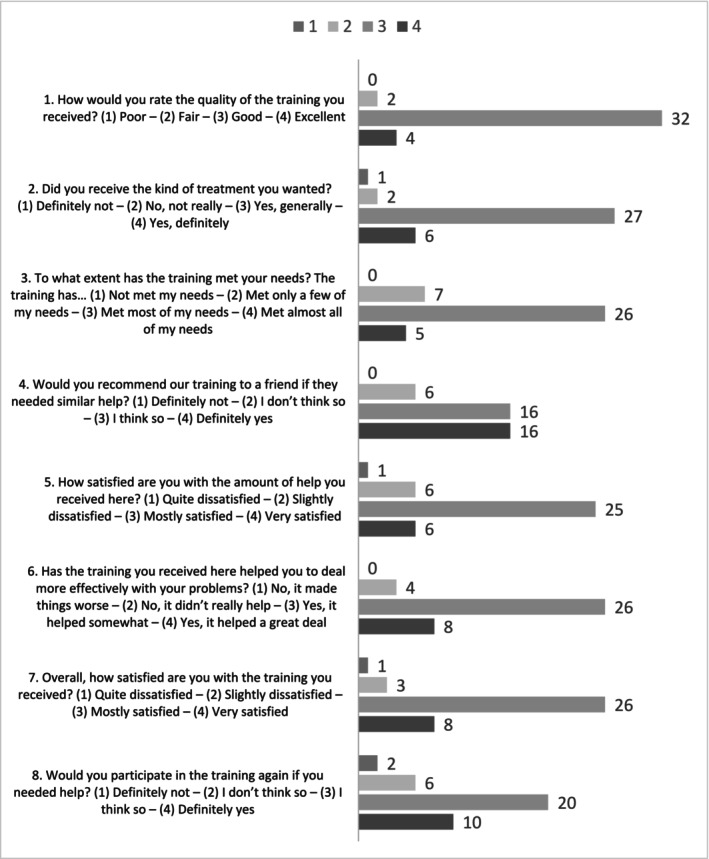
Results on satisfaction (Fragebogen zur Messung der Patientenzufriedenheit; ZUF‐8).

Figure 1 shows the results for the Items of the ZUF‐8. As satisfaction was measured post‐training, only the completers filled out the questionnaire. The training reached a mean sum score of 24.29 out of 32 points (SD = 3.70, *n* = 38). A majority of the completers (89%) were satisfied overall with the training (Item 7). Of the completers, 65.8% did not report any negative effects during or after the training. A total of 34.2% of the completers reported at least one side effect related to the training, including emotional distress, increased decision‐making difficulties and feelings of dependency. Some also reported interpersonal issues with the therapist, such as feeling hurt by comments or pressured to do certain exercises.

### 3.3 Preliminary Results Related to Effectiveness

Results of the fixed effects of LMM are displayed in Table 2. Full LMM results with all covariates can be found in the Supporting Information.

**TABLE 2:** Results of the linear mixed‐effects model (fixed effects).

Dependent variable	Predictor	Estimate	Std. error	*t*‐Value	*p*
Depression (QIDS‐SR)	T0 (Intercept)	10.65	5.02	2.12	0.039*
	T1	−0.25	0.70	−0.36	0.717
	T2	1.20	0.80	1.49	0.136
Depression (QIDS‐C)					
	T0 (Intercept)	15.20	5.05	3.01	0.004**
	T1	0.14	0.71	0.2	0.841
	T2^a^	−2.68	1.68	−1.59	0.117
Mania (ASRM)					
	T0 (Intercept)	2.19	2.16	1.01	0.315
	T1	−0.93	0.47	−1.99	0.047*
	T2	0.59	0.54	1.11	0.269
Mania (YMRS)					
	T0 (Intercept)	2.36	3.46	0.68	0.500
	T1	−1.20	0.65	−1.86	0.069
	T2^a^	−2.04	1.47	−1.38	0.170
Quality of Life (WHO‐QOL_BREF)					
	T0 (Intercept)	40.81	13.83774	2.95	0.005**
	T1	0.63	1.96	0.32	0.747
	T2	2.85	2.27	1.26	0.214
Metacognitions (MCQ‐30 Sum score)					
	T0 (Intercept)	14.92	2.55	5.85	0.000 ***
	T1	−0.60	0.43	−1.39	0.170
	T2	−0.72	0.52	−1.39	0.169
POS (MCQ‐30 Subscale)					
	T0 (Intercept)	15.27	3.16	4.84	0.000***
	T1	−1.67	0.57	−2.90	0.005**
	T2	−0.52	0.69	−0.76	0.451
NEG (MCQ‐30 Subscale)					
	T0 (Intercept)	14.96	5.25	2.85	0.006**
	T1	−0.57	0.700	−0.81	0.421
	T2	−1.69	0.84	−1.99	0.051
CES (MCQ‐30 Subscale)					
	T0 (Intercept)	13.46	4.02	3.35	0.002**
	T1	0.34	0.63	0.55	0.584
	T2	−0.15	0.75	−0.21	0.838
Dysfunctional Attitudes (DAS‐A and DAS‐B)					
	T0 (Intercept)	117.52	35.41	3.32	0.002**
	T1	3.56	3.82	0.93	0.355
	T2	8.64	4.64	1.86	0.067
Psychosocial functioning (MINI‐ICF)					
	T0 (Intercept)	3.28	0.94	3.50	0.001**
	T1	−0.44	0.15	−2.99	0.004**
	T2	−0.17	0.20	−0.83	0.414
Self‐management (FERUS)					
	T0 (Intercept)	227.70	33.34	6.83	0.000***
	T1	4.31	6.10	0.71	0.482
	T2	−3.54	9.61	−0.37	0.715
Mindfulness (FFA)					
	T0 (Intercept)	28.54	6.73	4.24	0.000***
	T1	0.43	1.05	0.41	0.683
	T2	3.26	1.24	2.62	0.011*

Abbreviations: ASRM = Altman Self‐Rating Mania Scale, C = Clinician‐Rated, CSC = Cognitive Self‐Consciousness, DAS = Dysfunctional Attitude Scale, FERUS = Fragebogen zur Erfassung von Ressourcen und Selbstmanagementfähigkeiten, FFA = Freiburger Fragebogen zur Achtsamkeit, MCQ30 = Metacognition Questionnaire, NEG = Negative Beliefs about Uncontrollability and Danger of Worry; POS = Positive Beliefs about Worrying, QIDS = Quick Inventory of Depressive Symptoms, SR = Self‐Rated, YMRS = Young Mania Rating Scale.

^a^ T2 estimates for QIDS‐C and YMRS should be interpreted with caution due to small sample size (*n* = 5).

* *p* < 0.05.

** *p* < 0.01.

*** *p* < 0.001.

#### 3.3.1 Depression

To analyse changes in both the depression scores, random‐intercept fixed‐slope models were used. Regarding QIDS‐SR, allowing for random slopes did not significantly improve model fit (χ^2^ = 4.96, *p* = 0.08). The assumptions of the LMM were evaluated and approximately met, though some heteroscedasticity in the fixed effects was observed. Log‐transforming the response variable did not resolve this issue. While these deviations may slightly affect model robustness, the results are interpreted with caution. Random intercept variance was 13.47 (SD = 3.67), and residual variance was 9.99 (SD = 3.16), indicating within‐individual variability that was not explained by covariates. For QIDS‐C, only five datapoints were available at follow‐up as the other participants were not able to come in for a clinical interview. This led to highly unbalanced data, which made it impossible to estimate a random slope for time and limited the reliability of follow‐up effects due to low statistical power and potential bias from non‐random missingness. As displayed by the fixed effects, the two depression scores did not change in a significant amount over the course of the study.

#### 3.3.2 Mania

Mania scores (ASRM and YMRS) were analysed using a random‐intercept fixed‐slope model as adding a random slope did not improve model fit (χ^2^ = 0.13, *p* = 0.93) for the ASRM and follow‐up data for the YMRS were insufficient. Results of ASRM indicated a significant decrease in scores from baseline to 1 week after training (T1); however, scores were already low at baseline due to inclusion criteria, suggesting a possible floor effect. Random intercept variance was 2.05 (SD = 1.43) and residual variance was 4.50 (SD = 2.12), reflecting high within‐participant variability, which could not be explained by any time‐invariant covariates that were added to the model. Model robustness was affected by slight non‐linearity of fixed effects, likely due to the highly skewed distribution of the response variable.

#### 3.3.3 Quality of Life

A random‐intercept fixed‐slope model was used to analyse changes in quality of life scores. Adding a random slope did not improve model fit (χ^2^ = 0.812, *p* = 0.67). Random intercept variance was 77.21 (SD = 8.79), and residual variance was 67.47 (SD = 8.21). None of the covariates accounted for unexplained variance. Assumptions of the LMM were met, and the model provided an adequate fit for the data.

#### 3.3.4 Metacognitions

Changes in MCQ‐30 subscale scores were analysed using random‐intercept fixed‐slope models as allowing for random slopes did not improve model fit in any of the models (sum score: χ^2^ = 0.83, *p* = 0.66; PB: χ^2^ = 1.75, *p* = 0.416; CSC: χ^2^ = 1.35, *p* = 0.508; NB: χ^2^ = 2.36, *p* = 0.307). Random intercept variance was 1.97 (SD = 1.41) for the sum score, 2.60 (SD = 1.61) for PB, 5.57 (SD = 2.36) for CSC and 11.35 (SD = 3.37) for NB. Residual variance was 3.62 (SD = 1.90) for sum score, 6.47 (SD = 2.54) for PB, 7.67 (SD = 2.77) for CSC and 9.51 (SD = 3.08) for NB. Model assumptions were met. For sum score and PB, gender was found to be statistically significant as a predictor, with female participants scoring lower. For the subscale PB, a score reduction was found from baseline to post‐treatment that was not maintained at follow‐up.

#### 3.3.5 Dysfunctional Attitudes

A random‐intercept fixed‐slope model was used to examine changes in dysfunctional attitudes. Including a random slope did not improve model fit (χ^2^ = 0.02, *p* = 0.91). No predictors significantly explained variability in dysfunctional attitudes. While scores increased from baseline to follow‐up by 8.64 points, this change was not statistically significant. Random intercept variance was 595.4 (SD = 24.40), and residual variance was 281.4 (SD = 16.78). Assumptions of the model were met.

#### 3.3.6 Psychosocial Functioning

Psychosocial functioning was analysed using a random‐intercept random‐slope model as adding a random slope significantly improved model fit (χ^2^ = 6.34, *p* = 0.042). Results indicated a decrease in ICF scores from baseline to T1; however, this reduction was not sustained at follow‐up (T2). Satisfaction was a significant predictor, with higher satisfaction associated with lower ICF scores. Current psychotherapy was negatively associated with ICF scores, suggesting a link between therapy and lower ICF. Session attendance was positively associated with ICF, indicating that participants with higher ICF scores attended more sessions. Random intercept variance was 0.68 (SD = 0.83), random slope variance was 0.13 (SD = 0.60) and residual variance was 0.34 (SD = 0.58). Model assumptions were met.

#### 3.3.7 Resources and Self‐Management

Scores of the FERUS were analysed using a random‐intercept random‐slope model, which was superior to a fixed‐slope model (χ^2^ = 13.04, *p* = 0.002). Age was a significant predictor, with higher age associated with lower FERUS scores. Random intercept variance was 309.1 (SD = 17.58), random slope variance was 390.7 (SD = 19.77) and residual variance was 527.1 (SD = 22.96). Model assumptions were met.

#### 3.3.8 Mindfulness

Changes in mindfulness (FFA scores) were analysed using a random‐intercept fixed‐slope model as allowing for random slopes did not improve model fit (χ^2^ = 0.745, *p* = 0.69). Follow‐up time points showed a positive effect on mindfulness, indicating an increase over time. Random intercept variance was 15.83 (SD = 3.98), and residual variance was 21.41 (SD = 4.63). Model assumptions were met.

## 4 Discussion

### 4.1 Main Findings

The study provides evidence for the feasibility of metacognitive training for BD in 48 psychiatric outpatients. The 8‐week program was designed to target cognitive biases that are typical for BD and therefore relieve symptoms and facilitate recognition of early warning signs of manic and depressive episodes. The results on satisfaction, attendance and dropout as markers of feasibility indicate that, among the completers, most of the participants were highly satisfied (89%) with the program and attended the majority of sessions (6.11 out of 8). The mean score of the CSQ (M = 24.29, SD = 3.70) seems similar to other psychotherapeutic group interventions (e.g., Scholl et al. 2021, M = 20.6, SD = 1.0; Saxon and Houghton 2007, M = 24.44, SD = 3.47). Notably, more than one‐third of the completers reported at least one negative side effect related to the intervention. While this proportion may appear high, it is comparable with rates observed in prior MCT studies (e.g., MCT‐D: 34% Dietrichkeit et al. 2021, MCT‐D/S: 71% Jelinek et al. 2021).

Although 20.83% of the participants dropped out of the study before completion, the dropout rate falls within the range reported in previous studies on MCT, which ranged from 0% (Kuokkanen et al. 2014; Simón‐Expósito and Felipe‐Castaño 2019; Fekete et al. 2022) to 43.48% (Briki et al. 2014; Liu et al. 2018) for psychosis and 6%–31% for depression (Jelinek et al. 2017). However, the dropout rate of the previous study on MCT‐Bipolar by Haffner et al. was lower (11.8%) (BPtK 2023). In our sample, no baseline variable predicted whether a participant would drop out of the study except for the MCQ‐30 subscale ‘Negative Beliefs about Uncontrollability and Danger of Worry’. More beliefs about the uncontrollability and danger of thoughts were associated with lower odds of dropping out. One possible explanation for this may be that participants who hold stronger negative beliefs about worry (e.g., “My worrying is dangerous for me”) are more motivated to try to manage their thoughts with metacognitive and mindfulness‐based strategies and are therefore more likely to adhere to the training.

Attendance, however, was not predicted by any baseline measures. This indicates that not attending may be explained by other variables that have nothing to do with attitudes towards the training, such as logistical or practical barriers. In this study, most of the participants stated that they did not attend due to sickness or work duties. These results suggest that the predictive value of demographic or clinical variables for attendance is rather low, which is consistent with the literature (Holdsworth et al. 2014). Although full attendance is vital in every study, preventing non‐attendance is challenging. Using reminder calls or letters to increase attendance has yielded mixed results (McLean et al. 2016). Offering online meetings may be a way to overcome practical treatment barriers such as long journeys. App‐based interventions may be useful to compensate for missed sessions. Although various smartphone‐based programs for BD have been developed in recent decades, their effect on reducing symptoms in BD remains to be established (Anmella et al. 2022).

The results of the LMMs give preliminary insights into changes over time regarding different outcome measures. It was found that the depression scores showed no significant change between baseline (T0) and post‐treatment (T1 and T2). This is in accordance with Haffner et al. (BPtK 2023) and also with other studies on group‐based intervention for BD. In a recent meta‐analysis, Tan et al. (2022) evaluated treatment outcomes of 11 studies of group‐based psychoeducation and group‐based CBT. Although it could be demonstrated that group‐based psychoeducation significantly reduced the relapse rate at post‐intervention, no significant results were found on improvement in depressive symptoms. Furthermore, group‐based CBT did not show any effect on relapse rate or depression scores. These results differ from studies with patients with unipolar depression, where group‐based CBT had a moderate effect on levels of depression up to 6 months after treatment (Feng et al. 2012). Also, MCT‐D led to larger improvements in depressive symptoms from baseline to follow‐up in comparison to an active control with moderate to strong effects (Jelinek et al. 2016). Questions remain regarding the differential effect of psychological interventions on patients with bipolar and unipolar disorder. Although symptoms of unipolar and bipolar depression overlap to a large degree in most domains, the two types differ in some cognitive styles. For example, bipolar depression is more associated with cognitive avoidance, preference for affiliation and less negative self‐esteem (Mansell et al. 2005). Because MCT‐Bipolar is an adapted version of D‐MCT, the content targeting depressive cognitive biases is similar in both versions. It may be that more adaptation to the characteristics of bipolar depression is needed to exhibit similar effects.

There was a small yet statistically significant reduction in the Altman Self‐Rating Mania Scale sum score from baseline to post‐treatment, though the effect disappeared from baseline to follow‐up. Given that the inclusion criteria limited participants to those with euthymic or depressive states, self‐rated baseline mania scores were already low. Consequently, the nearly one‐point reduction in mania was statistically significant yet not clinically relevant. Nonetheless, these findings highlight potential areas of interest for future research on group therapy for BD, particularly regarding how to effectively measure intervention effects on manic symptoms. In future studies on MCT‐Bipolar, researchers might address this issue by comparing the frequency of relapses or hospitalization (Colom, Vieta, Martinez‐Aran, et al. 2003; Isasi et al. 2010; Gomes et al. 2011). This may be achieved by monthly telephone interviews, which have proven to be a reliable and pragmatic method for gathering data on bipolar episodes (Castle et al. 2010; Revicki et al. 1997).

Impairments in psychosocial functioning significantly decreased during the training period, though these improvements were not maintained at follow‐up. These short‐term positive outcomes align with the initial study on MCT‐Bipolar by Haffner et al., in which an improvement in psychosocial functioning was observed using the Functioning Assessment Short Test (FAST) general score, demonstrating a large effect size post‐treatment (BPtK 2023). This supports the idea that MCT‐Bipolar may be an effective tool for addressing deficits in psychosocial functioning but needs adaptation to improve the sustainability of these effects. Future studies could explore strategies to reinforce the benefits of MCT‐Bipolar beyond the initial intervention period, such as booster sessions or additional support mechanisms, like smartphone applications (e.g., the COGITO App as a complementary tool for MCT‐D) (Moritz et al. 2024; Moritz et al. 2025).

Moreover, Haffner et al. found that individuals with greater baseline impairments experienced higher levels of improvement over the course of the intervention (BPtK 2023). This observation is further supported by the current study's LMM analysis, which revealed a high negative correlation between the slope and the intercept, indicating participants with initially higher impairment in psychosocial functioning showed greater reductions over time. These findings suggest that MCT‐Bipolar might be particularly beneficial for individuals with more severe baseline impairments, which implies the need to make the training practical for more impaired individuals as well.

Although no change in mindfulness scores was observed from pre‐ to post‐treatment (T0–T1), there was a significant increase from baseline to follow‐up (T0–T2). Each session began with a 10‐min mindfulness exercise. While some participants were already familiar with the concept of mindfulness, others were introduced to the mindfulness‐based approach for the first time during the training. It is possible that after the first encounter with mindfulness practices, participants continued practicing on their own after the training ended, leading to gradual improvement in their mindfulness skills over time.

Of the three subscales measured by the MCQ‐30, only the “Positive Beliefs about Worrying” (POS) subscale showed a decrease from baseline to post‐training. This subscale reflects beliefs regarding the usefulness of rumination in solving and preventing problems (e.g., “Worrying helps avoid future problems”). Consistent with the content of MCT‐Bipolar, participants reported fewer positive beliefs about the usefulness of worrying after the training. However, this effect did not persist at follow‐up, suggesting that maladaptive metacognitive beliefs are likely to remain stable over the long term and that MCT‐Bipolar was not able to change those beliefs sustainably. Consistent with findings from the prior study of MCT‐Bipolar, the current study did not demonstrate any improvement in global quality of life at post‐treatment, nor at follow‐up. Although Haffner and colleagues argued that the possibility of long‐term gains cannot be ruled out (BPtK 2023), as seen in the study on psychosis (Moritz et al. 2014), the results raise questions about the ability of MCT‐Bipolar to enhance perceived quality of life among individuals with BD.

### 4.2 Limitations

The study has several limitations that are worth consideration. First, the observational design without a control group limits the ability to draw causal inferences regarding the efficacy of MCT‐Bipolar. Moreover, patients' experiences outside of the training were not systematically monitored. Although medication and engagement in other psychotherapy were recorded at baseline, treatment changes were not assessed during the study period. Further, results are restricted to the specific sample and cannot be generalized to other samples. Notably, the data may be subject to self‐selection bias, as individuals who agree to participate are likely to differ systematically from those who do not, particularly in terms of motivation and treatment expectations, potentially leading to an underestimation of dropout rates (Sackett 1979).

Additional limitations regard the statistical procedures and measurement tools employed. Although LMMs offer advantages in handling missing data and modelling longitudinal change, they also come with assumptions and constraints. Notably, they require an adequate sample size with a sufficient number of repeated observations for each participant. The present study's small sample and its non‐negligible attrition‐related imbalance across time points likely reduced statistical power and increased the risk of Type II errors. Moreover, seven time‐invariant covariates were included in the models, which add to the model's complexity and make it vulnerable to misspecification.

Secondly, LMMs assume residuals of fixed and random effects to be normally distributed (Cohen et al. 2003), which was not the case for depression and mania in the current study. However, previous research (e.g., Schielzeth et al. 2020) has shown the robustness of mixed models even under violations. The suitability of the chosen measures presents an additional limitation. Most indicators were based on self‐report and thus susceptible to biases such as social desirability. Incorporating additional clinician‐rated measures would enhance the validity of future studies.

Thirdly, the null findings regarding change in depression scores may indicate that the use of the QIDS should be reconsidered for future research. This is supported by a recent study by Weiss et al. (2023), who expressed scepticism about the adequacy of QIDS as a measure of depression and its ability to detect treatment response. In comparison with other measures, the QIDS exhibited higher variance and standard errors, lower sensitivity due to compound items and unidimensional sum scoring, vagueness in the phrasing of scoring options for items and lack of focus on a core depression factor. For instance, the Beck Depression Inventory (BDI; Beck et al. 1996) was proven to be superior to the QIDS‐SR in studies measuring the effect of interventions on depression (Weiss et al. 2023). Overall, the null results may reflect limited statistical power, psychometric shortcomings of the instruments or the possibility that MCT‐Bipolar does not exert desired effects on the targeted constructs. Since the main objective of the study was to explore feasibility, a one‐arm observational design was chosen. To test the effectiveness of MCT‐Bipolar in the future, a randomized controlled trial should be applied.

## 5 Conclusion

In summary, this study supports MCT‐Bipolar as an acceptable and feasible group intervention for outpatients with BD in terms of patient satisfaction, dropout rates and attendance. Satisfaction was high, and the observed dropout and attendance rates were similar to the rates observed in other studies on MCT, indicating comparability of MCT‐Bipolar with already established MCT programs for other disorders. Preliminary findings on treatment efficacy found positive results on mindfulness and psychosocial functioning, although the effect on functioning diminished after 6 months. Digital components could additionally enhance the training and compensate for missed sessions (e.g., as in D‐MCT with the use of the app COGITO, uke.de/cogito). As a next step, the effectiveness of MCT‐Bipolar should be studied in the form of a randomized controlled trial.

## Funding

The authors have nothing to report.

## Conflicts of Interest

The authors declare no conflicts of interest. LJ is the developer of the MCT for depression (D‐MCT). SM is the developer of the MCT program.

## Supporting information


**Table S1:** Results of the Linear Mixed‐Effects Model (Quick Inventory of Depressive Symptomatology—Self‐Rating).
**Table S2:** Results of the Linear Mixed‐Effects Model (Quick Inventory of Depressive Symptomatology—Clinical Rating).
**Table S3:** Results of the Linear Mixed‐Effects Model (ASRM Sum Score).
**Table S4:** Results of the Linear Mixed‐Effects Model (YRSM Sum Score).
**Table S5:** Results of the Linear Mixed‐Effects Model (Quality of Life).
**Table S6:** Results of the Linear Mixed‐Effects Model (Metacognitions Sumscore).
**Table S7:** Results of the Linear Mixed‐Effects Model (Metacognitions: Positive Beliefs about Worry).
**Table S8:** Results of the Linear Mixed‐Effects Model (Metacognitions: Cognitive Self‐Consciousness).
**Table S9:** Results of the Linear Mixed‐Effects Model (Metacognitions: Negative Belief about Worry).
**Table S10:** Results of the Linear Mixed‐Effects Model (Dysfunctional Attitude Scale).
**Table S11:** Results of the Linear Mixed‐Effects Model (Psychosocial Functioning: MINI‐ICF).
**Table S12:** Results of the Linear Mixed‐Effects Model (Resources and Self‐Management: FERUS).
**Table S13:** Results of the Linear Mixed‐Effects Model (Mindfulness: FFA).

## Data Availability

The data generated and analysed during the current study are not publicly available due to privacy concerns.
