# Supplementary material for: Rethinking Cognitive Interventions in Bipolar Disorder: Feasibility and First Insights From Metacognitive Group Training (MCT‐Bipolar)
Source: Clin Psychol Psychother. 2026 Jun 7;33(3):e70294. doi: 10.1002/cpp.70294 (PMC13243341; doi:10.1002/cpp.70294)
Supplement: Supplementary file 1 — Table S1: Results of the Linear Mixed‐Effects Model (Quick Inventory of Depressive Symptomatology—Self‐Rating). Table S2: Results of the Linear Mixed‐Effects Model (Quick Inventory of Depressive Symptomatology—Clinical Rating). Table S3: Results of the Linear Mixed‐Effects Model (ASRM Sum Score). Table S4: Results of the Linear Mixed‐Effects Model (YRSM Sum Score). Table S5: Results of the Linear Mixed‐Effects Model (Quality of Life). Table S6: Results of the Linear Mixed‐Effects Model (Metacognitions Sumscore). Table S7: Results of the Linear Mixed‐Effects Model (Metacognitions: Positive Beliefs about Worry). Table S8: Results of the Linear Mixed‐Effects Model (Metacognitions: Cognitive Self‐Consciousness). Table S9: Results of the Linear Mixed‐Effects Model (Metacognitions: Negative Belief about Worry). Table S10: Results of the Linear Mixed‐Effects Model (Dysfunctional Attitude Scale). Table S11: Results of the Linear Mixed‐Effects Model (Psychosocial Functioning: MINI‐ICF). Table S12: Results of the Linear Mixed‐Effects Model (Resources and Self‐Management: FERUS). Table S13: Results of the Linear Mixed‐Effects Model (Mindfulness: FFA). [file CPP-33-e70294-s001.docx]

## SUPPLEMENTARY MATERIAL

## Table S1. Results of the Linear Mixed-Effects Model (Quick Inventory of Depressive Symptomatology – Self-Rating)

| Predictor | Estimate | Std. Error | t-value | p-value |
| --- | --- | --- | --- | --- |
| (Intercept) | 10.65 | 5.02 | 2.12 | .039 * |
| **Timepoints** |  |  |  |  |
| Post | -0.25 | 0.70 | -0.36 | .717 |
| Follow-Up | 1.20 | 0.80 | 1.49 | .136 |
| **Covariates** |  |  |  |  |
| Gender (female = 2) | 1.47 | 1.22 | 1.21 | .233 |
| Age | -0.02 | 0.05 | -0.37 | .716 |
| Satisfaction | -0.06 | 0.04 | -1.29 | .204 |
| Credibility | -0.08 | 0.14 | -0.59 | .561 |
| Expectancy | 0.06 | 0.12 | 0.44 | .659 |
| Psychotherapy (Yes = 2) | -0.16 | 1.16 | -0.14 | .888 |
| Attendance | 0.34 | 0.32 | 1.05 | .298 |

Notes:
- * p < .05, ** p <.01, ***p < .001
- Random effects included a random intercept for `record_id` (Variance = 13.47, SD = 3.67).
- Residual variance = 9.99, SD = 3.16.
- AIC = 2038.4, BIC = 2116.5.

## Table S2. Results of the Linear Mixed-Effects Model (Quick Inventory of Depressive Symptomatology – Clinical Rating)

| Predictor | Estimate | Std. Error | t-value | p-value |
| --- | --- | --- | --- | --- |
| (Intercept) | 15.20 | 5.05 | 3.01 | .004** |
| **Timepoints** |  |  |  |  |
| Post | 0.14 | 0.71 | 0.2 | .841 |
| Follow-Up | -2.68 | 1.68 | -1.59 | .117 |
| **Covariates** |  |  |  |  |
| Gender (female = 2) | 1.54 | 1.20 | 1.28 | .208 |
| Age | -0.07 | 0.05 | -1.31 | .197 |
| Satisfaction | -0.07 | 0.04 | -1.58 | .122 |
| Credibility | -0.11 | 0.14 | -0.83 | .409 |
| Expectancy | -0.04 | 0.13 | -0.33 | .743 |
| Psychotherapy (Yes = 2) | -1.33 | 1.15 | -1.16 | .254 |
| Attendance | 0.21 | 0.33 | 0.63 | .532 |

Notes:
- * p < .05, ** p <.01, ***p < .001
- Random effects included a random intercept for `record_id` (Variance = 9.27, SD = 3.05).
- Residual variance = 9.88, SD = 3.15.
- AIC = 533.1.4, BIC = 563.1

**Table S3. Results of the Linear Mixed-Effects Model (ASRM Sum Score)**

| Predictor | Estimate | Std. Error | t-value | p-value |
| --- | --- | --- | --- | --- |
| (Intercept) | 2.19 | 2.16 | 1.01 | .315 |
| **Timepoints** |  |  |  |  |
| Post | -0.93 | 0.47 | -1.99 | .047 * |
| Follow-Up | 0.59 | 0.54 | 1.11 | .269 |
| **Covariates** |  |  |  |  |
| Gender (female = 2) | 0.11 | 0.52 | 0.22 | .828 |
| Age | -0.03 | 0.02 | -1.17 | .247 |
| Satisfaction | -0.00 | 0.02 | -0.02 | .984 |
| Credibility | 0.08 | 0.06 | 1.44 | .157 |
| Expectancy | -0.01 | 0.05 | -0.11 | .913 |
| Psychotherapy (Yes = 2) | 0.15 | 0.49 | 0.32 | .754 |
| Attendance | -0.11 | 0.14 | -0.77 | .443 |

Notes:
- * p < .05, ** p <.01, ***p < .001
- Random effects included a random intercept for `record_id` (Variance = 2.05, SD = 1.43).
- Residual variance was Variance = 4.50, SD = 2.12.
- AIC = 1707.1, BIC = 1785.3.

**Table S4. Results of the Linear Mixed-Effects Model (YRSM Sum Score)**

| Predictor | Estimate | Std. Error | t-value | p-value |
| --- | --- | --- | --- | --- |
| (Intercept) | 2.36 | 3.46 | 0.68 | .500 |
| **Timepoints** |  |  |  |  |
| Post | -1.20 | 0.64 | -1.86 | .069 |
| Follow-Up | -2.04 | 1.47 | -1.39 | .170 |
| **Covariates** |  |  |  |  |
| Gender (female = 2) | -0.06 | 0.81 | -0.08 | .936 |
| Age | -0.03 | 0.04 | -0.76 | .451 |
| Satisfaction | -0.02 | 0.03 | -0.85 | .401 |
| Credibility | 0.14 | 0.09 | 1.45 | .145 |
| Expectancy | -0.14 | 0.08 | -1.77 | .084 |
| Psychotherapy (Yes = 2) | 0.39 | 0.78 | 0.50 | .620 |
| Attendance | 0.40 | 0.23 | 1.76 | .084 |

Notes:
- * p < .05, ** p <.01, ***p < .001
- Random effects included a random intercept for `record_id` (Variance = 2.31, SD = 1.52).
- Residual variance was Variance = 8.17, SD = 2.86.
- AIC = 488.6, BIC = 518.6

**Table S5. Results of the Linear Mixed-Effects Model (Quality of Life)**

| Predictor | Estimate | Std. Error | t-value | p-value |
| --- | --- | --- | --- | --- |
| (Intercept) | 40.81 | 13.84 | 2.95 | .005 ** |
| **Timepoints** |  |  |  |  |
| Post | 0.63 | 1.96 | 0.32 | .747 |
| Follow-Up | 2.85 | 2.27 | 1.26 | .214 |
| **Covariates** |  |  |  |  |
| Gender (female = 2) | -4.71 | 3.35 | -1.41 | .167 |
| Age | 0.08 | 0.15 | 0.56 | .578 |
| Satisfaction | 0.18 | 0.12 | 1.56 | .127 |
| Credibility | 0.56 | 0.37 | 1.50 | .141 |
| Expectancy | -0.09 | 0.34 | -0.28 | .782 |
| Psychotherapy (Yes = 2) | 2.09 | 3.24 | 0.65 | .521 |
| Attendance | -0.92 | 0.95 | -0.97 | .337 |

Notes:
- * p < .05, ** p <.01, ***p < .001
- Random effects included a random intercept for `record_id` (Variance = 77.21, SD = 8.787).
- Residual variance = 67.47, SD = 8.21.
- AIC = 791.6, BIC = 823.0

## Table S6. Results of the Linear Mixed-Effects Model (Metacognitions Sumscore)

| Predictor | Estimate | Std. Error | t-value | p-value |
| --- | --- | --- | --- | --- |
| (Intercept) | 14.92 | 2.55 | 5.85 | < .001 *** |
| **Timepoints** |  |  |  |  |
| Post | -0.56 | 0.43 | -1.39 | .171 |
| Follow-Up | -0.72 | 0.52 | -1.39 | .169 |
| Covariates |  |  |  |  |
| Gender (female = 2) | -1.24 | 0.60 | -2.09 | .043 * |
| Age | -0.04 | 0.03 | -1.38 | .175 |
| Satisfaction | -0.01 | 0.02 | -0.38 | .705 |
| Credibility | 0.02 | 0.07 | 0.35 | .731 |
| Expectancy | 0.05 | 0.06 | 0.82 | .418 |
| Psychotherapy (Yes = 2) | 0.41 | 0.58 | 0.71 | .479 |
| Attendance | 0.14 | 0.17 | 0.82 | .414 |

Notes:
- * p < .05, ** p <.01, ***p < .001
- Random effects included a random intercept for `record_id` (Variance = 1.973, SD = 1.405).
- Residual variance = 3.620, SD = 1.903.
- AIC = 506.7, BIC = 538.9.

## Table S7. Results of the Linear Mixed-Effects Model (Metacognitions: Positive Beliefs about Worry )

| Predictor | Estimate | Std. Error | t-value | p-value |
| --- | --- | --- | --- | --- |
| (Intercept) | 15.27 | 3.16 | 4.84 | < .000 *** |
| **Timepoints** |  |  |  |  |
| Post | -1.67 | 0.57 | -2.91 | .005 ** |
| Follow-Up | -0.52 | 0.69 | -0.76 | .451 |
| **Covariates** |  |  |  |  |
| Gender (female = 2) | -2.20 | 0.74 | -2.95 | .004 ** |
| Age | -0.02 | 0.03 | -0.47 | .639 |
| Satisfaction | 0.01 | 0.03 | 0.27 | .788 |
| Credibility | -0.05 | 0.08 | -0.56 | .577 |
| Expectancy | 0.05 | 0.08 | 0.63 | .529 |
| Psychotherapy (Yes = 2) | 0.02 | 0.71 | -0.02 | .982 |
| Attendance | -0.28 | 0.21 | -1.35 | .181 |

Notes:
- * p < .05, ** p <.01, ***p < .001
- Random effects included a random intercept for `record_id` (Variance = 2.60, SD = 1.61).
- Residual variance = 6.47, SD = 2.54.
- AIC = 562.2 BIC = 594.3.

## Table S8. Results of the Linear Mixed-Effects Model (Metacognitions: Cognitive Self-Consciousness)

| Predictor | Estimate | Std. Error | t-value | p-value |
| --- | --- | --- | --- | --- |
| (Intercept) | 13.46 | 4.02 | 3.349 | .002 ** |
| **Timepoints** |  |  |  |  |
| Post | 0.34 | 0.63 | 0.55 | .584 |
| Follow-Up | -0.15 | 0.76 | -0.21 | .838 |
| **Covariates** |  |  |  |  |
| Gender (female = 2) | -1.35 | 0.95 | -1.42 | .162 |
| Age | -0.05 | 0.04 | -1.30 | .199 |
| Satisfaction | 0.01 | 0.03 | 0.09 | .930 |
| Credibility | 0.05 | 0.11 | 0.49 | .623 |
| Expectancy | 0.20 | 0.10 | 2.09 | .042 * |
| Psychotherapy (Yes = 2) | 0.57 | 0.91 | 0.62 | .547 |
| Attendance | 0.10 | 0.26 | 0.37 | .712 |

Notes:
- * p < .05, ** p <.01, ***p < .001
- Random effects included a random intercept for `record_id` (Variance = 5.58, SD = 2.36).
- Residual variance = 7.67, SD = 2.77.
- AIC = 595.5, BIC = 627.7.

## Table S9. Results of the Linear Mixed-Effects Model (Metacognitions: Negative Belief about Worry)

| Predictor | Estimate | Std. Error | t-value | p-value |
| --- | --- | --- | --- | --- |
| (Intercept) | 14.96 | 5.25 | 2.85 | .006 ** |
| **Timepoints** |  |  |  |  |
| Post | -0.57 | 0.70 | -0.81 | .421 |
| Follow-Up | -1.68 | 0.85 | -1.99 | .051 |
| **Covariates** |  |  |  |  |
| Gender (female = 2) | -0.30 | 1.25 | -0.24 | .810 |
| Age | -0.03 | 0.05 | -0.59 | .560 |
| Satisfaction | -0.03 | 0.04 | -0.64 | .526 |
| Credibility | 0.07 | 0.14 | 0.52 | .606 |
| Expectancy | -0.11 | 0.13 | -0.82 | .415 |
| Psychotherapy (Yes = 2) | 0.74 | 1.20 | 0.61 | .543 |
| Attendance | 0.65 | 0.34 | 1.90 | .062 |

Notes:
- * p < .05, ** p <.01, ***p < .001
- Random effects included a random intercept for `record_id` (Variance = 11.35, SD = 3.37).
- Residual variance was Variance = 9.51, SD = 3.08.
- AIC = 643.1, BIC = 666.3.

## Table S10. Results of the Linear Mixed-Effects Model (Dysfunctional Attitude Scale)

| Predictor | Estimate | Std. Error | t-value | p-value |
| --- | --- | --- | --- | --- |
| (Intercept) | 117.52 | 35.41 | 3.32 | .002 ** |
| **Timepoints** |  |  |  |  |
| Post | 3.56 | 3.82 | 0.93 | .355 |
| Follow-Up | 8.64 | 4.64 | 1.86 | .067 |
| **Covariates** |  |  |  |  |
| Gender (female = 2) | -5.75 | 8.51 | -0.68 | .503 |
| Age | 0.55 | 0.37 | 1.49 | .144 |
| Satisfaction | 0.43 | 0.30 | 1.42 | .161 |
| Credibility | -0.27 | 0.96 | -0.28 | .782 |
| Expectancy | -0.26 | 0.87 | -0.30 | .770 |
| Psychotherapy (Yes = 2) | 2.44 | 8.14 | 0.30 | .766 |
| Attendance | -0.22 | 2.27 | -0.10 | .923 |

Notes:
- * p < .05, ** p <.01, ***p < .001
- Random effects included a random intercept for `record_id` (Variance = 595.4, SD = 24.40).
- Residual variance = 281.4, SD = 16.78.
- AIC = 1020.5, BIC = 1052.7.

## Table S11. Results of the Linear Mixed-Effects Model (Psychosocial Functioning: MINI-ICF)

| Predictor | Estimate | Std. Error | t-value | p-value |
| --- | --- | --- | --- | --- |
| (Intercept) | 3.28 | 0.94 | 3.50 | .001 ** |
| **Timepoints** |  |  |  |  |
| Post | -0.44 | 0.15 | -2.99 | .004 ** |
| Follow-Up | -0.17 | 0.20 | -0.83 | .41410 |
| **Covariates** |  |  |  |  |
| Gender (female = 2) | 0.01 | 0.22 | 0.06 | .956 |
| Age | 0.01 | 0.01 | 0.82 | .415 |
| Satisfaction | -0.02 | 0.01 | -2.04 | .048 * |
| Credibility | 0.01 | 0.02 | 0.29 | .776 |
| Expectancy | -0.04 | 0.02 | -1.97 | .055 |
| Psychotherapy (Yes = 2) | -0.48 | 0.21 | -2.24 | .030 * |
| Attendance | 0.13 | 0.07 | 2.04 | .046 * |

Notes:
- * p < .05, ** p <.01, ***p < .001
- Random effects included a random intercept for `record_id` (Variance = 0.68, SD = 0.83) and a random slope (Variance = 0.13, SD = -365), r = - 0.85
- Residual variance = 0.34, SD = 0.58.
- AIC = 282.2, BIC = 319.3.

## Table S12. Results of the Linear Mixed-Effects Model (Resources and Self-Management: FERUS)

| Predictor | Estimate | Std. Error | t-value | p-value |
| --- | --- | --- | --- | --- |
| (Intercept) | 227.70 | 33.34 | 6.83 | < .001 *** |
| **Timepoints** |  |  |  |  |
| Post | 4.31 | 6.10 | 0.71 | .482 |
| Follow-Up | -3.54 | 9.61 | -0.37 | .715 |
| **Covariates** |  |  |  |  |
| Gender (female = 2) | 0.81 | 7.94 | 0.10 | .919 |
| Age | -0.83 | 0.34 | -2.42 | .020 * |
| Satisfaction | 0.34 | 0.28 | 1.22 | .229 |
| Credibility | 0.42 | 0.89 | 0.47 | .643 |
| Expectancy | 0.43 | 0.81 | 0.54 | .593 |
| Psychotherapy (Yes = 2) | 1.75 | 7.58 | 0.23 | .819 |
| Attendance | -1.20 | 2.15 | -0.56 | .580 |

Notes:
- * p < .05, ** p <.01, ***p < .001

- Random effects included a random intercept for `record_id` (Variance = 309.1 SD = 17.58) and a random slope (Variance = 390.7, SD = 19.77), r = - 0.13
- Residual variance = 527.1, SD = 22.96.
- AIC = 1082.0, BIC = 1119.6.

## Table S13. Results of the Linear Mixed-Effects Model (Mindfulness: FFA)

| Predictor | Estimate | Std. Error | t-value | p-value |
| --- | --- | --- | --- | --- |
| (Intercept) | 28.54 | 6.73 | 4.24 | < .001 *** |
| **Timepoints** |  |  |  |  |
| Post | 0.43 | 1.05 | 0.41 | .683 |
| Follow-Up | 3.26 | 1.24 | 2.62 | .011 * |
| **Covariates** |  |  |  |  |
| Gender (female = 2) | -0.06 | 1.59 | -0.04 | .970 |
| Age | 0.01 | 0.07 | 0.15 | .880 |
| Satisfaction | 0.06 | 0.06 | 1.11 | .274 |
| Credibility | 0.00 | 0.18 | 0.01 | .992 |
| Expectancy | 0.14 | 0.16 | 0.87 | .388 |
| Psychotherapy (Yes = 2) | 0.81 | 1.53 | 0.53 | .600 |
| Attendance | -0.14 | 0.44 | -0.31 | .761 |

Notes:
- * p < .05, ** p <.01, ***p < .001

- Random effects included a random intercepts for ‘record_id’ (Variance = 15.83, SD = 3.98)

- Residual variance = 21.41 (SD = 4.63).

- AIC = 713.0, BIC = 745.3.
